# Molecular allergens drive risk stratification and immunotherapy in Hymenoptera venom allergy

**DOI:** 10.1016/j.waojou.2025.101128

**Published:** 2025-10-09

**Authors:** Enrico Scala, Valeria Villella, Damiano Abeni, Mauro Giani, Emma Cristina Guerra, Maria Locanto, Giorgia Meneguzzi, Lia Pirrotta, Donato Quaratino, Alessandra Zaffiro, Elisabetta Caprini, Riccardo Asero, Lorenzo Cecchi, Danilo Villalta, Valerio Pravettoni

**Affiliations:** a Fondazione Luigi Maria Monti, IDI-IRCCS, Rome, Italy; b Ambulatorio di Allergologia, Clinica San Carlo, Paderno Dugnano, Milan, Italy; c Allergy and Clinical Immunology Unit, San Giovanni di Dio Hospital, Florence, Italy; d S.C. di Immunologia e Allergologia di Laboratorio, PO S. Maria degli Angeli, Pordenone, Italy; e Department of Internal Medicine, Fondazione IRCCS Ca' Granda Ospedale Maggiore Policlinico, Milan, Italy

**Keywords:** Hymenoptera venom allergy, Component-resolved diagnostics, Venom immunotherapy, Panallergens, Api m 1, Ves v 1, Ves v 5, Precision allergy

## Abstract

**Background:**

Hymenoptera venom allergy is a major cause of life-threatening anaphylaxis. Molecular diagnostics have improved the characterisation of sensitisation patterns, although the clinical role of cross-reactive allergens such as DPPIV and hyaluronidases remains unclear.

**Objective:**

To define IgE sensitisation to venom components, examine associations with clinical phenotypes, and assess the immunological impact of venom immunotherapy (VIT) in an Italian cohort.

**Methods:**

In this monocentric study, 378 patients with documented Hymenoptera adverse reactions underwent extract-based and component-resolved diagnostics. A prospective subset (n = 113) was followed during VIT. Commercial venom extracts were characterised by inhibition assays.

**Results:**

Systemic reactions occurred in 36% of patients, predominantly males, while large local reactions were more common in females. Sensitisation to *Vespula* spp. (52%) and *Polistes dominula* (48%) exceeded *Apis mellifera* (26%). Api m 1/3/10 and Ves v 5/Pol d 5 were the most relevant allergens. Multivariate analysis identified IgE to Api m 1, Api m 3, Ves v 1, and Ves v 5 as independent predictors of systemic reactions. Mixed-effect models revealed a progressive decline of species-specific IgE during VIT, with an earlier progressive reduction of Ves v 1 and Ves v 5 and a delayed decrease of Api m 1, while IgE to panallergens remained stable.

**Conclusion:**

Molecular diagnostics refine risk stratification and monitoring of VIT. Species-specific allergens provide reliable clinical markers, whereas panallergens help distinguish true double sensitisation from cross-reactivity. VIT induces a progressive but differential reduction in specific IgE and confers protection, supporting precision allergy care.

## Introduction

### Background and rationale

Hymenoptera venom allergy (HVA) continues to pose a significant clinical challenge, given its potential to trigger reactions ranging from large local to severe, life-threatening anaphylaxis.^1^^,^^2^ The most implicated species in Mediterranean area—*Apis mellifera, Vespula* spp.*, Polistes dominula*, and *Vespa crabro*—release complex venoms containing a wide array of allergenic components.^3^^,^^4^ Advances in component-resolved diagnostics (CRD) have markedly improved our ability to dissect IgE sensitisation profiles at the molecular level.^5^^,^^6^ However, the clinical implications of certain allergens—especially cross reactive allergens such as dipeptidyl-peptidase IV and hyaluronidases—remain incompletely defined, limiting their translational utility in risk stratification and therapeutic guidance.^7^, ^8^, ^9^, ^10^

Vespid venom allergy is elicited by a variety of species worldwide, with *Vespula* spp. representing the predominant culprits in the Northern Hemisphere, *Polistes* spp. being more relevant in the United States and Mediterranean Europe, and additional *Polistinae* such as *Polybia* spp. gaining importance in South America. Allergies to hornet stings (*Vespa* and *Dolichovespula* spp.) are also frequent and may be rising due to the spread of invasive species such as *Vespa velutina nigrithorax*,^11^ whose venom is up to now not commercially available in Italy. At the molecular level, phospholipase A1 (Ves v 1, Pol d 1) and antigen 5 (Ves v 5, Pol d 5) represent the most relevant allergens across vespid venoms,^12^, ^13^, ^14^, ^15^, ^16^, ^17^, ^18^, ^19^, ^20^, ^21^, ^22^, ^23^ providing high diagnostic sensitivity in yellow jacket venom and *Polistes dominula* venom allergy. However, their extensive cross-reactivity within *Vespoidea* species limits their reliability as marker allergens for defining primary sensitisation.^3^^,^^12^^,^^19^^,^^23^, ^24^, ^25^

In contrast, the European honeybee (*Apis mellifera*) is the most prominent species worldwide eliciting venom allergy, and its allergenic proteins have therefore been extensively characterized.^26^, ^27^, ^28^ The main honeybee venom (HBV) allergens are currently available for CRD, although their accessibility varies depending on the assay platform and the country. Among them, Api m 1, Api m 3, Api m 4, and Api m 10 have emerged as pivotal marker allergens for identifying primary HBV sensitisation,^8^^,^^16^^,^^29^, ^30^, ^31^ thereby enabling CRD to accurately differentiate between HBV and vespid venom allergy and to improve diagnostic sensitivity in patients with complex sensitisation profiles.^8^^,^^16^^,^^27^^,^^32^, ^33^, ^34^, ^35^

As a national referral centre, our Institute has established a longitudinal cohort of patients with venom allergy, offering a robust framework to investigate the molecular underpinnings of sensitisation and their correlation with clinical phenotypes and immunotherapeutic outcomes. This study builds on that foundation to refine our understanding of how molecular profiles shape clinical trajectories in HVA.

### Objectives

The primary aim of this study was to characterize IgE sensitisation profiles to hymenoptera venom components and assess their clinical relevance in a well-defined Italian cohort. We hypothesised that distinct molecular sensitisation patterns are associated with clinical severity and response to immunotherapy,^36^ paving the way for more precise and personalised management strategies in venom allergy.^34^

## Methods

### Study design and setting

This was a single-center observational study with prospective interventional design and retrospective (2015–2023) data collection, conducted at the outpatient allergy clinics of Fondazione Luigi Maria Monti | Istituto Dermopatico dell’Immacolata – IRCCS (IDI-IRCCS) in Rome, Italy, between January 2015 and December 2023. The retrospective phase focused on the molecular characterization of sensitisation profiles and their clinical correlates, whereas the prospective component evaluated the immunological and clinical effects of venom immunotherapy (VIT) over time (Table S1).

Patients were consecutively enrolled, and those undergoing VIT were followed with annual assessments. VIT was initiated using a cluster protocol consisting of 4 injections on the first day at 20–30-min intervals, followed by weekly dose escalations with aqueous extracts or depot preparations, in accordance with the manufacturers’ instructions. ^37^

The main study objectives were to:^1^ quantify the prevalence of large local and systemic reactions, including potential sex-related differences;^2^ characterize molecular sensitisation patterns across different Hymenoptera species, distinguishing species-specific markers from cross-reactive allergens;^3^ investigate associations between molecular IgE profiles and the risk of systemic reactions; and^4^ evaluate the immunological effects of VIT on allergen-specific IgE reactivity, including inhibition assays to infer the allergenic composition and potency of therapeutic extracts.

### Study population and measurements

Eligible patients had a documented history of adverse reactions to Hymenoptera stings, including large local reactions (LLRs) and systemic reactions. They were retrospectively identified through the TD-Synergy® Laboratory Information System (Siemens Healthcare Diagnostics s.r.l., Milan, Italy) and a dedicated institutional electronic database that systematically records demographic, clinical, and laboratory data. Inclusion required evidence of sensitisation to at least 1 venom (*Apis mellifera*, V*espula* spp., *Polistes dominula*, or *Vespa crabro*) based on extract-based and/or molecular testing. Patients who had undergone dual VIT or had known mast cell disorders were excluded from statistical analyses. Ethical approval was obtained (IDI-IRCCS, 494/1), and all participants provided written informed consent.

Clinical reactions were classified as either LLRs—defined as swelling exceeding 10 cm, increasing over 24–48 h and persisting >24 h in the absence of systemic symptoms^38^ — or systemic reactions, extending beyond the sting site and including generalized urticaria, respiratory distress, angioedema, dizziness, or loss of consciousness. Sting reactions were graded according to internationally accepted severity scales.^39^

Data were extracted from clinical records and laboratory results, including skin prick tests, intradermal tests, baseline tryptase levels, and serum allergen-specific IgE assays to crude venom extracts and recombinant allergens available on the market. Allergen-specific IgE antibodies to whole venom extracts and individual molecular components were quantified using the ImmunoCAP™ system (Thermo Fisher Scientific, Uppsala, Sweden), according to the manufacturer's instructions. The panel included both native and recombinant allergens, covering species-specific markers (eg, Api m 1, Api m 3, Api m 10), vespid components (Ves v 1, Ves v 5, and Pol d 5), and cross-reactive molecules such as Api m 2 and Api m 5. The ImmunoCAP assay for Api m 10 became commercially available in 2015. Results were expressed in kUA/L, with values ≥ 0.35 kUA/L considered positive. However, in view of the increased analytical sensitivity of current assays (detection limit 0.10 kUA/L),^40^ venom-specific IgE levels between 0.10 and 0.35 kUA/L were also scored as positive in patients with low total IgE (<10 kUA/L). Testing was performed as part of routine diagnostic evaluation and longitudinal follow-up during immunotherapy.

In selected cases, serial IgE measurements and *in vitro* inhibition assays with commercial venom preparations were performed to track changes in sensitisation profiles over time and to assess the molecular composition and allergenic potency of therapeutic extracts.

For inhibition experiments, 100 μl of pooled sera from 15 sensitized individuals with documented allergy to *Apis mellifera* and European *Vespula* spp. were incubated overnight with an equal volume of either phosphate-buffered saline (PBS, negative control) or commercial aqueous VIT extracts. The extracts tested were: (i) bee venom from Anallergo (ANA, Scarperia e San Piero, Florence, Italy) and Aquagen SQ (ALK Abelló, Hørsholm, Denmark); and (ii) *Vespula* spp. venom from ANA or Allergy Therapeutics (AT, Settimo Milanese, Milan, Italy). Inhibition with ALK was not performed for *Vespula* as no aqueous preparation was available.

The following day, 40 μl aliquots of adsorbed and control sera were analyzed using the ImmunoCAP™ system according to the manufacturer's instructions. For Apis mellifera, both crude venom extracts (ANA and ALK) and the molecular components Api m 1, Api m 3, and Api m 10 were tested. For *Vespula* spp. and *Polistes dominula*, crude venom extracts (ANA and AT) as well as the molecular components Ves v 1, Ves v 5, and Pol d 5 were evaluated. In addition, all samples—including adsorbed and control sera—were analyzed for IgE reactivity to Api m 2 (hyaluronidase) and Api m 5 (dipeptidyl peptidase IV), representing the 2 major cross-reactive allergens.

### Statistical analysis

Descriptive statistics were used to summarise demographic, clinical, and sensitisation data. Categorical variables were compared using chi-squared or Fisher's exact test; continuous variables were analyzed using t-tests or ANOVA as appropriate. Multivariable logistic regression was performed to identify independent associations between molecular sensitisation and clinical outcomes, while adjusting for age, sex, and CCD sensitisation. Correlations between IgE responses to different allergens were assessed with Spearman's rank correlation. A p-value <0.050 was considered statistically significant. These analyses were conducted with SPSS software (version 27.0, IBM Corp., Armonk, NY, USA).

Time trends in molecular allergens levels were modelled using mixed-effects analyses with random intercepts, accounting for between-subject variability; all models were performed with the Jamovi statistical software (The jamovi project - 2024 v2.6. Retrieved from https://www.jamovi.org.).

## Result

### Study population and baseline characteristics

Between January 2015 and December 2023, a total of 66,377 patients were evaluated for allergic disorders at our centre. Among these, 378 individuals (0.57%) reported adverse reactions to Hymenoptera stings and were included in the study cohort. LLRs were observed in 64% of cases, occurring significantly more frequently in females (p < 0.01), while systemic reactions were reported in 36% of patients and were significantly more common among males (p < 0.01) (Table 1).

**Table 1:** Demographic, clinical, and sensitisation characteristics of the study population.

	**Total**		**Reaction type**			
	**Population**		*LLRs*		*Systemic*	
	**N**	**%**	**N**	**%**	**N**	%
**Overall**	378	*100%*	241	*64%*	137	*36%*
**Sex (M |F)**	187 | 191	*49% | 51%*	82 | 156∗	*34% | 66%*	102 | 35∗	*74% | 26%*
**Age (mean + STD)**	49 + 18		49 + 19		50 + 15	
**Total IgE (geometric mean)**	83		71		99	
**Tryptase (ng/ml) (mean** ± **ES)**	5.6 ± 0.7		3.1 ± 0.5		8.1 ± 1.8∗	

∗P < 0.05.

### Sensitisation patterns and clinical phenotypes

Overall, sensitisation to vespid venoms was more prevalent than to honeybee venom. Specific IgE responses were detected in 52% of patients for *Vespula* spp., 48% for *Polistes dominula*, and 34% for *Vespa crabro*, compared to only 26% for *Apis mellifera* (Table 1). Tryptase levels were 3.1 ± 0.5 ng/mL, median 2.1 ng/mL in subjects with large local reactions, whereas higher levels were observed in cases of systemic reactions (8.1 ± 1.8 ng/mL, median 5.1 ng/mL; P < 0.05).

**Fig. 1:**
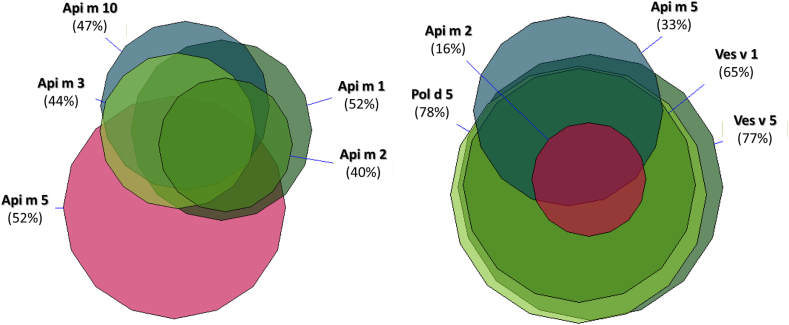
Molecular sensitisation profiles in patients positive to Apis mellifera whole venom extract (A) and in those sensitized to Vespula spp. extract (B).

Molecular profiling revealed that among individuals with IgE reactivity to *Apis mellifera* extract, 52% were sensitized to Api m 1 and 47% to Api m 10 (Table 2). After excluding those monosensitised to the cross-reactive panallergen Api m 5, these rates rose to 74% for Api m 1, 61% for Api m 3, and 75% for Api m 10. In a subset of 25 allergic beekeepers, Api m 1 was recognised in 64% of cases, while both Api m 3 and Api m 10 were positive in 56%. Notably, approximately half of the *Apis*-reactive individuals were sensitized to dipeptidyl peptidase IV (DPPIV), rather than to genuine marker allergens (Fig. 1).

**Table 2:** Comparison of sensitisation profiles obtained with whole venom extracts (*Apis mellifera*, *Vespula* spp., *Polistes dominula*, and *Vespa crabro*) and with the currently available molecular components in the 378 individuals included in the study. Values ≥ 0.35 kUA/L were considered positive (≥0.1 kUA/L for molecular components in patients with total IgE <10 kU/L). “N” indicates the number of patients, while “%” refers to the proportion of positive results for both the extract and the corresponding molecular component

			Molecular components								
			rApi m 1 Phospholipase A2	rApi m 2 Hyaluronidase	rApi m 3 Acid phosphatase	rApi m 5 DPPIV	rApi m 10 Icarapin	rPol d 5 Antigen 5	rVes v 1 Phospholipase A1B	rVes v 5 Antigen 5	MUXF3 CCD Marker
Whole venom extracts	Apis mellifera (98)	N	**51**	**39**	**43**	**51**	**46**	**58**	**48**	**58**	**42**
		*%*	*52%*	*40%*	*44%*	*52%*	*47%*	*59%*	*49%*	*59%*	*43%*
	Vespula spp (195)	N	**37**	**31**	**33**	**65**	**38**	**149**	**126**	**151**	**45**
		*%*	*19%*	*16%*	*17%*	*33%*	*19%*	*76%*	*65%*	*77%*	*23%*
	Polistes dominula (181)	N	**31**	**28**	**29**	**60**	**32**	**142**	**117**	**138**	**42**
		*%*	*17%*	*15%*	*16%*	*33%*	*18%*	*78%*	*65%*	*76%*	*23%*
	Vespa Crabro (128)	N	**29**	**26**	**27**	**58**	**25**	**110**	**95**	**107**	**45**
		*%*	*23%*	*20%*	*21%*	*45%*	*20%*	*86%*	*74%*	*84%*	*35%*

Among patients sensitized to vespids, over 75% showed IgE reactivity to species-specific molecular markers, including Ves v 5 (*Vespula*) and Pol d 5 (*Polistes*). In those reactive to *Vespa crabro*, Ves v 1 and Ves v 5 were detected in 75% and 84% of cases, respectively. In contrast, Api m 5 was less frequently recognised among vespid-allergic individuals (33–45%) than among those sensitized to *Apis mellifera* venom (approximately 50%). Sensitisation to hyaluronidase was observed in 40% of bee venom–reactive patients and in fewer than 20% of vespid-reactive individuals, while CCD-specific IgE was detected in 23–43% of cases, with the highest prevalence among *Apis* venom–sensitized patients. DPPIV also emerged as a likely source of non-genuine IgE reactivity in vespid-allergic patients lacking IgE to Ves v 1, Ves v 5, and Pol d 5. By contrast, hyaluronidase sensitisation appeared evenly distributed among individuals reactive to genuine venom components.

Mono-sensitisation to a single venom source was confirmed in 44 individuals for *Apis mellifera* (including 6 with IgE to vespid molecules but not to whole vespid extracts), 4 for *Polistes*, 13 for *Vespula*, and none for *Vespa crabro*. As a result, nearly 84% of patients displayed co-sensitisation to multiple venom sources (Table S2).

Sensitisation to most molecular components was significantly associated with systemic reactions in univariate analyses (p < 0.01, Table 3). To account for potential confounding factors, we subsequently performed multivariate logistic regression models adjusted for age, sex, and CCD reactivity. In these adjusted models, only IgE reactivity to Api m 1, Api m 3, Ves v 1, and Ves v 5 remained independently associated with severe systemic reactions.

**Table 3:** Associations between molecular sensitisation profiles and clinical outcomes (local vs. systemic reactions). Univariate analyses (left panel) report crude odds ratios (OR) with 95% confidence intervals (CIs), while multivariate models (right panel) show adjusted ORs after correction for age, sex and CCD reactivity. P values indicate the statistical significance of the associations

		**Overall**		**LLRs**		**Systemic Reactions**					
**Variable**	**Level**	**N**	**%**	**N**	**%**	**N**	**%**	**OR (95% C.I.)**	**P-value**	**ORadj (95% C.I.)**	**P-value**
**rApi m 1**	No	294	84%	207	93%	87	68%	1		1	
*PLA A2*	Yes	57	16%	16	7%	41	32%	6.1 (3.2–11.4)	<0.001	**8.4 (1.8–38.3)**	0**.006**
**rApi m 2**	No	302	87%	200	91%	102	80%	1		1	
*HYAL*	Yes	45	13%	19	9%	26	20%	2.7 (1.4–5.1)	0.002	1.1 (0.3–4.2)	0.884
**rApi m 3**	No	281	86%	195	96%	86	69%	1		1	
*ACPase*	Yes	47	14%	9	4%	38	31%	9.6 (4.4–2.7)	<0.001	**6.4 (1.4–30.2)**	0**.018**
**rApi m 5**	No	263	77%	189	88%	74	58%	1		1	
*DPPIV*	Yes	78	23%	25	12%	53	42%	5.4 (3.1–9.3)	<0.001	0.8 (0.3–2.0)	0.654
**rApi m 10**	No	282	83%	193	91%	89	70%	1		1	
*Icarapin*	Yes	58	17%	20	9%	38	30%	4.1 (2.3–7.5)	<0.001	0.6 (0.2–1.9)	0.384
**rPol d 5**	No	161	49%	136	70%	25	19%	1		1	
*Antigen 5*	Yes	167	51%	57	30%	110	81%	1.5 (6.2–17.9)	<0.001	2.1 (0.6–6.9)	0.233
**rVes v 1**	No	184	58%	146	78%	38	28%	1		1	
*PLA A1B*	Yes	136	43%	40	22%	96	72%	9.2 (5.5–15.4)	<0.001	**2.9 (1.2–6.9)**	0**.015**
**rVes v 5**	No	152	47%	128	68%	24	18%	1		1	
*Antigen 5*	Yes	173	53%	61	32%	112	82%	9.8 (5.7–16.7)	<0.001	**8.6 (2.5–30.0)**	0**.001**
**MUXF3**	No	172	87%	67	92%	105	88%	1		1	
*CCD*	Yes	25	13%	6	8%	19	12%	2.0 (,7-5.3)	0.148	0.9 (0.2–4.3)	0.937

### Prospective evaluation of venom immunotherapy and longitudinal molecular monitoring

A subgroup of 113 patients was enrolled in the prospective phase and initiated venom immunotherapy (VIT) with commercially available extracts licensed in Italy. Among them, 37 patients (33%) received Apis mellifera venom (25 treated with ALK and 12 with ANA), while 76 patients (67%) were treated with Vespula spp. venom (30 with ALK, 21 with AT, and 25 with ANA). Treatment selection was based on careful identification of the culprit insect through detailed clinical history—particularly in beekeepers or patients able to accurately recognize the offending insect—supported by skin testing (both skin prick and intradermal) and *in vitro* diagnostics using extracts and molecular components, including specific IgE inhibition (with both extracts and single molecules, according to CAP inhibition procedures, including adsorption with MUXF3 to exclude CCD-mediated signals) and/or basophil activation tests when indicated. All patients received single-venom immunotherapy, and those with known mast cell disorders were excluded.

During the 4-year follow-up, all 113 patients (notably, 25/37 sensitized to Apidae were beekeepers and 26/76 sensitized to Vespidae were farmers or living in rural settings) experienced at least 1 re-sting by either Apidae or Vespidae. No systemic reactions were reported, and LLRs occurred only in the first year of VIT, affecting approximately 5% of patients (6 in total: 5 after *Apis mellifera* stings and 1 after a vespid sting). All patients, having been repeatedly trained not only in the use of rescue medications but also in insect recognition, were able to report whether the stinging insect belonged to Apidae or Vespidae. However, in the absence of independent validation, a precise identification at the species level could not be confirmed.

In patients undergoing venom immunotherapy, longitudinal linear mixed-effect analisys models revealed distinct trajectories of specific IgE responses to individual Apis mellifera and *Vespula* components. For honeybee allergens, rApi m 1 showed a significant overall time effect (p = 0.007), with reductions becoming evident only at year 3 (p = 0.035) and year 4 (p = 0.023) compared with baseline. rApi m 5 also demonstrated a global time effect (p = 0.016), but significant reduction was restricted to the late comparison between year 1 and year 4 (p = 0.011). In contrast, rApi m 2, rApi m 3, and rApi m 10 did not exhibit significant changes across the 4 years. For vespid allergy, both Ves v 1 and Ves v 5 displayed robust declines over time. rVes v 1 showed a significant overall effect (p < 0.001), with decreases becoming evident from year 2 onwards (p = 0.046 at T2, p = 0.002 at T3, p = 0.003 at T4), while rVes v 5 exhibited an early and sustained reduction already detectable after the first year (p = 0.034) and persisting through years 2–4 (all p < 0.001) (Table 4).

**Table 4:**
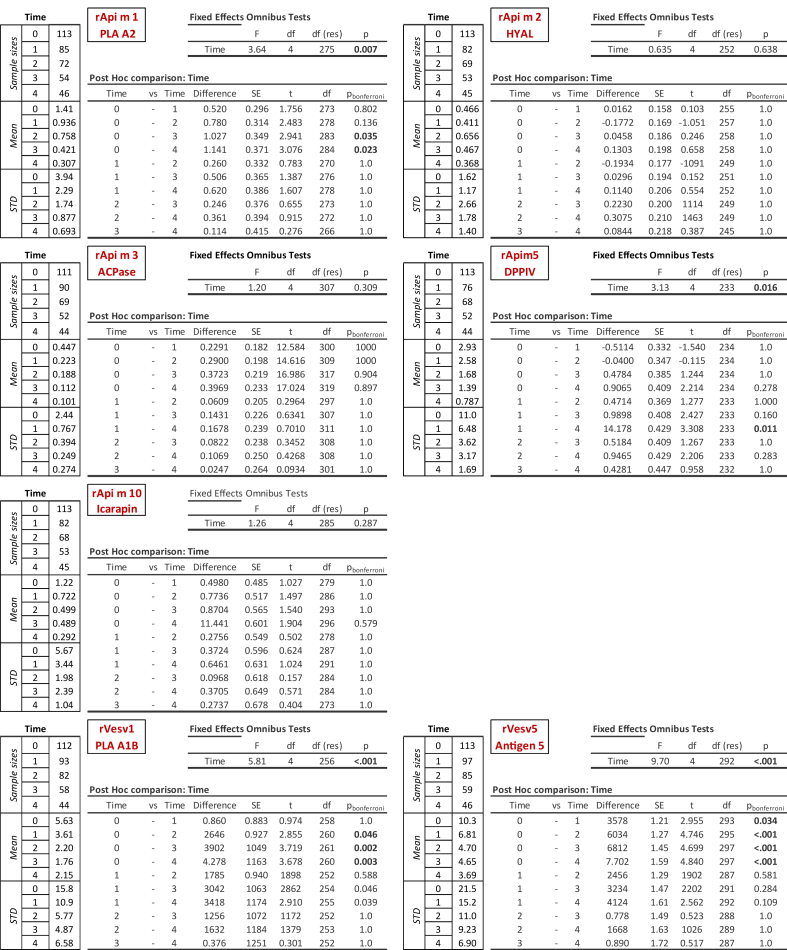
Specific IgE levels (kU/L) to molecular components were measured at baseline (T0) and after 1–4 years (T1–T4) of venom immunotherapy in *Apis mellifera* and *Vespula* spp. allergy. Mean ± SD and sample sizes are reported. Linear mixed-effects models showed significant decreases for Ves v 1, Ves v 5, and Api m 1 (all p < 0.01), a late decline for Api m 5 (p = 0.011), while Api m 2, Api m 3, and Api m 10 remained unchanged. Between-subject variability explained a substantial proportion of variance (ICC range: 0.668–0.962)

Unexpectedly, 4 patients undergoing bee VIT developed *de novo* sensitisation to Api m 5 after the first year. All were beekeepers, suggesting that natural re-stings may have contributed, though epitope spreading or minor allergens in the therapeutic extracts cannot be excluded. Across all molecules, mixed model analyses highlighted substantial between-subject variability, with high intraclass correlation coefficients (ICC), indicating that individual differences contributed significantly to overall variance in molecular IgE levels.

#### Inhibition assays

**Fig. 2:**
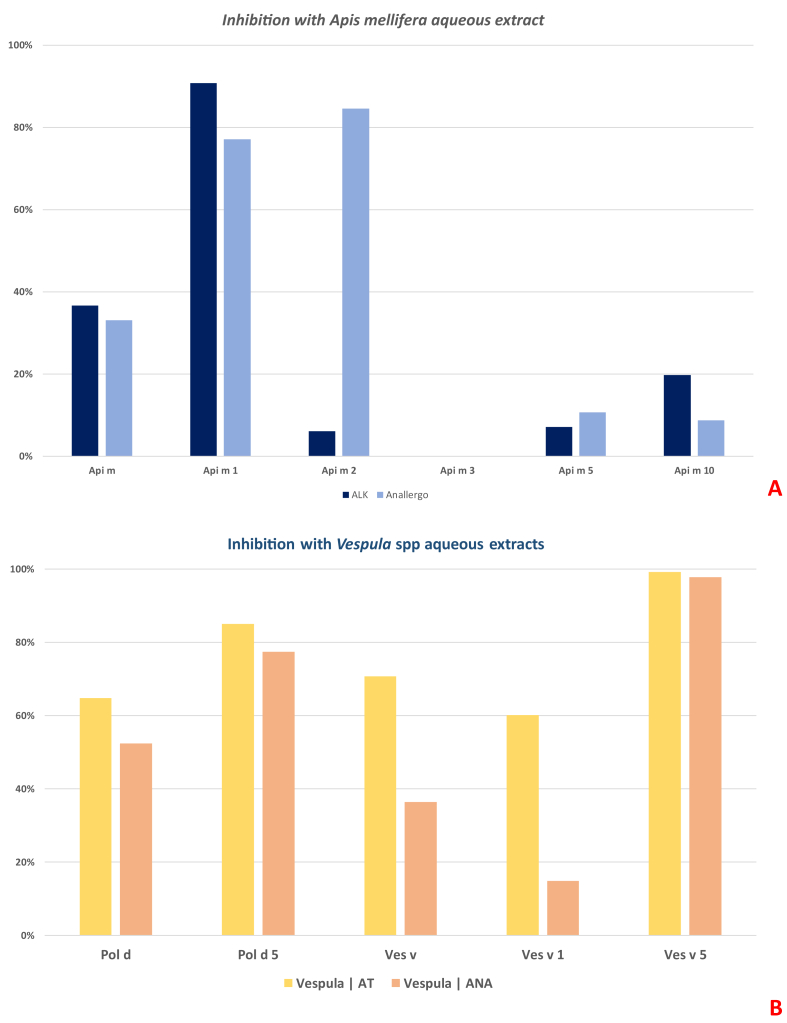
*In vitro* IgE inhibition assays with commercial venom immunotherapy extracts. Pooled sera from sensitized patients were tested for IgE binding to honeybee (A) and vespid (B) molecular allergens. Inhibition assays were performed using available aqueous VIT extracts, as indicated.

*In vitro* inhibition assays were performed using pooled sera from sensitized individuals and commercially available aqueous VIT extracts. For *Apis mellifera*, IgE binding to Api m 1 was inhibited by both ALK and ANA extracts, whereas inhibition of Api m 2 was observed only with ANA, suggesting the presence of hyaluronidase in its formulation. No inhibition was detected for Api m 3, and only marginal inhibition was observed for Api m 5 and Api m 10 (Fig. 2A).

For vespid venoms, both AT and ANA extracts effectively inhibited IgE binding to Ves v 5 and Pol d 5 (inhibition with ALK was not performed, as the aqueous extract for Vespula was not available). In contrast, inhibition of Ves v 1 was partial (∼60%) with the AT extract and minimal (<20%) with the ANA preparation, highlighting substantial differences in molecular composition among the tested products (Fig. 2B).

## Discussion

### Principal findings and interpretation

This study provides a real-world overview of molecular sensitisation profiles in hymenoptera venom allergy across an extended observational period. Sensitisation to vespid venoms clearly predominated over *Apis mellifera*, consistent with Mediterranean exposure patterns.^41^

LLRs were more frequent in females, whereas systemic reactions were associated with male sex and specific IgE profiles. Ves v 1 and Ves v 5 (*Vespula*), along with Api m 1 and Api m 3 (*Apis mellifera*), emerged as independent predictors of systemic reactions, reinforcing their role as biomarkers for risk stratification. 7, 8, 9^,^^20^^,^^31^^,^^33^^,^^42^, ^43^, ^44^ The high rate of co-sensitisation (84%) further underscores the value of molecular diagnostics in distinguishing genuine sensitisation from cross-reactivity. ^43^^,^^45^ However, the absence of Pol d 1 still limits the discrimination of *Polistes allergy* from *Vespula* or other vespids.^19^

Our data also emphasise the importance of Api m 1, Api m 3, and Api m 10 as diagnostic markers of *Apis mellifera allergy*.^31^^,^^43^^,^^46^^,^^47^ Only 52% of patients sensitized to *Apis mellifera* whole extracts showed IgE to these molecules, indicating that nearly half of extract-based positives may not represent true allergy. ^43^^,^^45^ Hence, component-resolved diagnostics are critical to reduce misclassification and guide management. ^42^ This diagnostic gap highlights the heterogeneity of bee venom sensitisation and supports the inclusion of additional allergens (Api m 2, Api m 3, Api m 4, Api m 5, Api m 10) in patients with systemic reactions but negative Api m 1. ^5^^,^^10^^,^^34^^,^^36^^,^^48^ Yet, the current panel excludes Api m 4,^10^ Api m 6,^49^ Api m 7–9, Api m 11,^50^ and Api m 12, remaining far from comprehensive.

### Implications for immunotherapy and precision allergy care

Longitudinal mixed-effect analyses showed that venom immunotherapy (VIT) does not uniformly affect all allergens but induces distinct kinetic patterns. Ves v 1 and Ves v 5 declined early and persistently, supporting their role as dominant vespid sensitizers and biomarkers of treatment response, although definitive conclusions on 10.13039/100019904VIT efficacy would require direct comparison between responders and non-responders. Api m 1 and Api m 5 decreased more gradually, while Api m 2, Api m 3, and Api m 10 remained stable, suggesting selective IgE modulation by VIT. Clinically, serial monitoring of Ves v 5, Ves v 1, and Api m 1 may provide practical guidance for evaluating treatment efficacy and tailoring its duration.

Reported VIT protection rates range from 71 to 75% in honeybee allergy and 73–100% in wasp allergy,^51^^,^^52^ with partial protection in nearly all patients.^52^^,^^53^ In our cohort, only mild large local reactions (∼5%) occurred during the first year, likely reflecting the use of purified depot preparations rather than aqueous extracts, although the latter may be advantageous in select individuals. However, the limited sample size—especially among bee venom–treated patients—precludes firm conclusions. Moreover, repeated natural re-stings, common in beekeepers, may attenuate IgE decline during VIT, as noted by Pravettoni et al.^54^

These findings reinforce the need for high-resolution molecular profiling at baseline and longitudinally, to refine patient stratification and monitoring. ^21^^,^^34^^,^^55^ They also call for better standardisation and molecular characterisation of venom extracts to optimise efficacy and minimise incomplete desensitisation.^5^^,^^16^^,^^33^^,^^47^^,^^48^^,^^55^

### Study limitations and methodological considerations

Limitations include potential bias from retrospective screening, pooled sera in inhibition assays, and exclusion of patients on double-venom immunotherapy. The ratio of specific to total IgE was not analyzed, though exploratory data suggest value. Finally, outcomes were assessed following natural re-stings, as sting challenges are not allowed in our Institute. This circumstance could have contributed to the lack of non-responders observed. Nevertheless, the integration of prospective data, molecular profiling, and extract characterisation strengthens the robustness of our findings.

### Generalisability and future directions

Our Southern European cohort limits direct extrapolation to regions where Apis mellifera predominates. ^41^ Future studies should include larger prospective cohorts, incorporate sting challenge outcomes, and evaluate VIT failure rates. Molecular monitoring during VIT could serve as an early surrogate marker of efficacy, enabling adaptive therapy. Finally, discrepancies in extract composition highlight the need for harmonised regulatory standards and improved allergen standardisation across manufacturers.

### Conclusions and perspectives

This study reaffirms the essential role of molecular diagnostics in hymenoptera venom allergy, both for accurate diagnosis and for guiding immunotherapy. Identification of Ves v 1, Ves v 5, Api m 1, and Api m 3 as independent risk markers supports risk stratification. Stable IgE responses to panallergens and differential VIT effects across venom sources highlight the need for allergen-specific monitoring. Expanding access to molecular diagnostics and improving venom extract fidelity are key steps towards precision allergy care, promising better risk assessment, treatment optimisation, and patient outcomes.

## Disclosure of the use of generative AI and AI-assisted technologies

Nothing to disclose.

## Institutional review board statement

The research was conducted in accordance with ethical standards outlined in the World Medical Association Declaration of Helsinki. The study protocol underwent review and approval by the IDI–IRCCS's committee, with approval number IDI-IRCCS CE | 494/1.

## Data availability statement

The data supporting the findings of this study are available upon request from the corresponding author. However, please note that the data are not publicly available due to privacy or ethical restrictions.

## Author contributions

E.S., V.V., and E.C. conducted the experiments and collected the data; E.S., MG, ECG, ML, GM, LP, DQ, and AZ recruited the patients; E.S. and D.A. performed the statistical analysis; E.S. conceived the study and assisted in data interpretation and wrote the first draft of the manuscript; V.P., L.C., D.V. and R.A. assisted in data interpretation. All authors reviewed, edited, and approved the final manuscript. All authors consent to publication, confirm that this manuscript is original, has not been published previously, is not under consideration for publication elsewhere, and has not been submitted to a preprint server. All authors have read and agreed to the published version of the manuscript.

## Funding

This study was supported by the Ministero della Salute “Progetto Ricerca Corrente” of the 10.13039/501100003196Italian Ministry of Health, Rome, Italy.

## Declaration of competing interest

ES has received consultant arrangements and participated in speakers' bureaus for Dasit and Thermo Fisher Scientific. The remaining authors declare that they have no relevant conflicts of interest.
