# Supplementary material for: Molecular allergens drive risk stratification and immunotherapy in Hymenoptera venom allergy
Source: World Allergy Organ J. 2025 Oct 9;18(10):101128. doi: 10.1016/j.waojou.2025.101128 (PMC12546951; doi:10.1016/j.waojou.2025.101128)
Supplement: Multimedia component 1 [file mmc1.docx]

**Table S1. Summary of study design, population, and analytical approach**

| **Domain** | **Description** |
| --- | --- |
| **Study design** | Retrospective observational study with prospective interventional extension (2015–2023, IDI-IRCCS Rome). |
| **Setting** | Outpatient allergy clinics; annual follow-up during VIT. Cluster build-up schedule: four administrations on Day 1 (20–30 min intervals), followed by weekly injections for 8 weeks (aqueous, depot, or sequential extracts). |
| **Population** | Patients with documented adverse reactions to hymenoptera stings (large local or systemic). Identified through institutional databases. Evidence of sensitisation to *Apis mellifera, Vespula spp., Polistes dominula*, or *Vespa crabro* required for inclusion. Patients receiving dual VIT excluded from statistical analysis. |
| **Clinical outcomes** | Large local reactions: swelling >10 cm, increasing over 24–48 h and lasting >24 h without systemic symptoms. Systemic reactions: manifestations beyond sting site (urticaria, angioedema, respiratory difficulty, dizziness, syncope). Severity graded according to internationally accepted scales. |
| **Laboratory measures** | Skin prick and intradermal tests; serum tryptase; allergen-specific IgE (whole extracts and molecular components via ImmunoCAP™). Components included Api m 1, Api m 3, Api m 10, Ves v 1, Ves v 5, Pol d 5, Api m 2, Api m 5. Selected cases underwent longitudinal IgE monitoring and in vitro inhibition assays with commercial venom extracts. |
| **Bias control** | Standardised diagnostic protocols, stringent inclusion criteria, pre-specified analyses, sensitivity analyses for robustness. |
| **Statistical analysis** | Descriptive statistics; χ² or Fisher’s exact test for categorical variables; t-test/ANOVA for continuous variables. Multivariable logistic regression adjusting for age, sex, and CCD sensitisation. Spearman’s correlation for IgE profiles. Significance threshold: p < 0.05. Analyses performed with SPSS v27.0. |
